# IslandViewer update: improved genomic island discovery and visualization

**DOI:** 10.1093/nar/gkt394

**Published:** 2013-05-15

**Authors:** Bhavjinder K. Dhillon, Terry A. Chiu, Matthew R. Laird, Morgan G. I. Langille, Fiona S. L. Brinkman

**Affiliations:** ^1^Department of Molecular Biology and Biochemistry, Simon Fraser University, Burnaby, British Columbia V5A 1S6, Canada and ^2^Department of Biochemistry and Molecular Biology, Dalhousie University, Halifax, Nova Scotia B3H 4R2, Canada

## Abstract

IslandViewer (http://pathogenomics.sfu.ca/islandviewer) is a web-accessible application for the computational prediction and analysis of genomic islands (GIs) in bacterial and archaeal genomes. GIs are clusters of genes of probable horizontal origin and are of high interest because they disproportionately encode virulence factors and other adaptations of medical, environmental and industrial interest. Many computational tools exist for the prediction of GIs, but three of the most accurate methods are available in integrated form via IslandViewer: IslandPath-DIMOB, SIGI-HMM and IslandPick. IslandViewer GI predictions are precomputed for all complete microbial genomes from National Center for Biotechnology Information, with an option to upload other genomes and/or perform customized analyses using different settings. Here, we report recent changes to the IslandViewer framework that have vastly improved its efficiency in handling an increasing number of users, plus better facilitate custom genome analyses. Users may also now overlay additional annotations such as virulence factors, antibiotic resistance genes and pathogen-associated genes on top of current GI predictions. Comparisons of GIs between user-selected genomes are now facilitated through a highly requested side-by-side viewer. IslandViewer improvements aim to provide a more flexible interface, coupled with additional highly relevant annotation information, to aid analysis of GIs in diverse microbial species.

## INTRODUCTION

Genomic islands (GIs) are most commonly defined as clusters of genes of probable horizontal origin in bacterial and archaeal genomes (1). These regions are of interest owing to their potential to confer rapid adaptation of the microbe (2,3). The first GIs identified were termed pathogenicity islands (4), conferring changes in pathogen virulence; however, subsequently multiple classes of GIs were identified that play a much broader role in the evolution of other traits including increased metabolic capacity and symbiosis (2,5). For this reason, researchers from a wide array of fields have an interest in predicting GIs in bacteria and archaea. From a medical perspective, GIs are of interest because they have been shown to disproportionately encode virulence factors (6), and can encode antimicrobial resistance and other adaptations that increase pathogen fitness and transmissibility. Researchers and public health workers need to rapidly characterize such regions during infectious disease outbreaks, as well as for more fundamental pathogen research. Islands conferring other metabolic adaptations are also of environmental and industrial interest (7). For example, a study by Tumapa *et al.* (8) has shown GIs are widespread in both clinical and environmental strains of the human pathogen *Burkholderia pseudomallei*, but are not significantly associated with disease-causing strains, instead performing a more general role in the metabolic fitness of this species. Therefore, predicting GIs in diverse microbial species, and understanding the types of genes present and their function in the overall system, has become of growing interest.

IslandViewer (9) was originally released in 2009 as the first web server aiding discovery of GIs in microbial genomes through the integration of the three most accurate GI prediction algorithms [as evaluated previously (10)]. This new release of IslandViewer contains significant changes that improve the efficiency of running GI predictions on user-specified sequences, helps avoid common user errors and incorporates additional user-requested annotation information plus a simple comparative genomics view to aid further investigation of GIs.

## ISLANDVIEWER METHODS

### GI prediction methodology

GIs possess many features that can be manipulated for computational prediction, such as differing sequence composition bias versus the host genome, flanking direct repeats or insertion near tRNA genes (1). Current algorithms are generally based either on (i) detecting sequence features and sequence composition differences (i.e. dinucleotide bias) or (ii) using comparative genomics to find unique regions compared with several related isolates. Many of these tools alone cannot predict all GIs in a given genome; thus, IslandViewer was developed to incorporate three distinct methods (10): SIGI-HMM (11) and IslandPath-DIMOB (12), two sequence composition-based methods and IslandPick (10), a comparative genomics-based method. Since this evaluation was performed, new GI prediction methods have been published such as GI-POP (13), GIST (14), GIHunter (15), EGID (16) and the Z-island method (17). GI-POP is the only other new tool available through a web interface to also provide visualization of predictions. However, it is specifically designed to predict GIs on draft genomes using a support vector machine classifier trained on data sets generated by IslandPick, with a reported slight loss of accuracy compared with our methods. IslandViewer remains a valuable web service for the prediction of GIs because it incorporates three methods using distinct complementary GI properties for detection, along with the ability to further customize GI prediction using the IslandPick comparative genomics-based method and its settings.

### Web server implementation

All IslandViewer web pages are written in PHP with Perl helper scripts to run dynamic processes. IslandViewer also relies on a recently upgraded MySQL server to store GI predictions. The set of available precomputed GI predictions are currently calculated on all complete microbial genomes downloaded every month from the National Center for Biotechnology Information using MicrobeDB (18). Over 1000 additional publicly available complete genomes have been precomputed for GIs since the first release of IslandViewer. Users may submit custom genome sequence data in GenBank or EMBL format, and results are stored in the database for a minimum of 1 month. All custom jobs submitted to the web server are run in parallel on a set of cluster nodes. GI prediction results are displayed to users in a variety of formats: circular genome plots highlighting the location of GIs are generated for every genome using Circos v0.62-1 (19), and interactively present gene annotations within GIs. This image can be customized to display predictions from one or all of the different algorithms. A summary table is provided with further annotation data about the actual sequences, genes and proteins encoded within each predicted GI region, also differentiated by prediction method. All of this information can be downloaded in multiple formats, including high-resolution versions of the images in SVG format for use in publications/presentations and simple parsing of data for storage in other databases if necessary.

## RECENT DEVELOPMENTS

### Improved management of custom genome analyses

IslandViewer allows users the option to upload their own genome of interest to run through the entire GI prediction pipeline. Since its initial release, the back end of this particular feature has been significantly improved, increasing its efficiency in processing data as well as providing better feedback to users about the most commonly encountered analysis problems. Some of these improvements were obvious, for example, the custom genome analysis pipeline has been modularized so that failures in one of the GI prediction tools do not cause the entire pipeline to fail. This was a major concern because occasional failures beyond our control in the third-party SIGI-HMM program could result in predictions from the other two methods not being reported. IslandViewer now also provides better format checking and feedback to users uploading input files for the custom genome analysis. The most common user-based issues, such as missing protein translations, are now checked for as the file is uploaded. Documentation providing more advice for high-quality GI analysis is also now provided. Hardware upgrades coupled with improved management and parallelization of jobs can now accommodate an increasing number of jobs as our user base grows. All of these changes greatly improve the user experience in running custom genomes through IslandViewer.

### Incorporation of external gene annotation data

**Figure 1.**
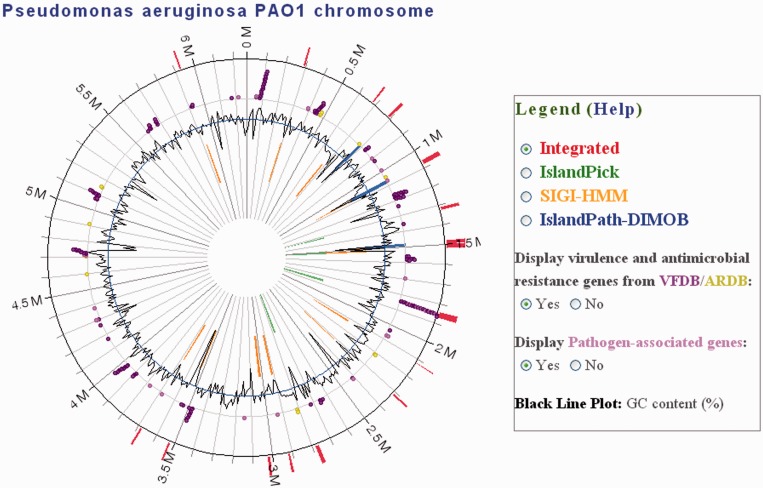
IslandViewer output image highlighting GI predictions for *Pseudomonas aeruginosa* PAO1. The circle represents a single chromosome, with red bars around the perimeter indicating the locations of all GI predictions across the three methods. Within the circle, GI predictions are differentiated by prediction method with IslandPath-DIMOB (blue), SIGI-HMM (orange) and IslandPick (green) all shown. Within the legend, specific predictors can be selected to view the results for just one method (and settings for manipulation are displayed when the IslandPick display is shown). The new virulence scatter plot, which can be toggled on/off, highlights in this case genes with an annotation in VFDB (purple), ARDB (yellow) and pathogen-associated genes (pink). Side-by-side views of multiple genomes are now possible.

GI investigations can be notably improved by integrating additional annotations associated with the genes in the islands. IslandViewer now incorporates gene annotations from two external databases: the Virulence Factor Database (VFDB) (20) and the Antibiotic Resistance Genes Database (ARDB) (21). As seen in Figure 1, the external annotations are visualized alongside the GI predictions in a scatter plot track around the genome, with VFDB-classified genes noted in purple and ARDB-classified genes marked in yellow. Users can now more easily identify GIs that are pathogenicity islands or antibiotic resistance islands using this new feature. At the time of publication, 1661 virulence factor classifications were mapped to proteins in IslandViewer from VFDB, while 956 proteins were classified as being involved in antibiotic resistance from the ARDB (complete genomes only). It should be emphasized that currently, only the actual genes in VFDB and the ARDB are noted in IslandViewer, rather than propagating annotations across many genomes using similarity-based approaches (which can be inaccurate owing to the contextual nature of virulence, for example). This ensures that IslandViewer continues to be a quality resource with accurate information. However, we are incorporating a pathogen-associated genes analysis previously computed (6) that identifies genes found only in pathogens in multiple genera, but not in any non-pathogen genome [see (6) for cutoffs used]. Such annotations are shaded in a lighter colour, and marked differently in the table, to ensure the user appreciates that they are similarity-based predictions, even though previous analyses indicated that these pathogen-associated genes are highly associated with ‘offensive’ (versus defensive) disease-causing capabilities. This pathogen-associated genes analysis will be further expanded and refined for a future release, reflecting the high interest in such pathogen-specific genes.

### Genome comparison viewer

Also new in IslandViewer is a ‘compare genomes’ feature, to visualize GI predictions across two genomes simultaneously. This new option displays predictions from two user-selected genomes side-by-side and provides access to download concatenated images for use in presentations or publications. This feature has been highly requested by users because closely related isolates are routinely compared and custom genome analyses are often compared with precomputed reference genomes. Note that methods for alignment of similar regions between genomes were investigated, but based on user feedback, a simple side-by-side comparison was deemed most useful because it is unbiased by any similarity measures and does not get complicated by large genome rearrangements. Usually highly similar genomes are displayed side-by-side, with the origin at the top of the circular chromosome, which orients the genome displays.

## DISCUSSION

The latest release of IslandViewer showcases various improvements to better facilitate the study of GIs in bacterial and archaeal genomes. These improvements include those most frequently demanded by users in both academia and public health labs. IslandViewer is now equipped for handling an increasing number of users and provides more feedback to ease the use of custom genome analyses. More information is now available to guide users through the various capabilities of IslandViewer. Furthermore, the ability to overlay external annotation data from relevant data sets as well as visualizing two genomes simultaneously in this new version of IslandViewer will prove to be invaluable for more rapid characterization of microbial genomes and their GIs.

Upcoming developments for IslandViewer include an expanded pathogen-associated genes analysis and the ability to predict GIs on draft genomes by accommodating contig boundaries in our existing prediction methods. GI-POP (13) has similar functionality; however, by expanding IslandViewer to handle draft genomes, users will be able to perform analysis in the context of multiple complementary methods and can further compare results with annotated reference genomes. In all, IslandViewer is an invaluable resource readily available through the web for the study of GIs in the Bacteria and Archaea, and the described improvements will further assist GI analysis, potentially expanding our knowledge of GIs and their role in key adaptive processes.

## FUNDING

Genome British Columbia/Genome Canada; the Simon Fraser University Community Trust Endowment Fund; Canadian Institutes for Health Research Bioinformatics Training Program scholarship (to B.K.D.); Canadian Institute for Health Research (Postdoctoral Fellowship Award to M.G.I.L.); the Michael Smith Foundation for Health Research (Scholar Award to F.S.L.B.). Funding for open access charge: Genome Canada.

*Conflict of interest statement.* None declared.

## References

[1] Langille MG, Hsiao WW, Brinkman FS (2010). Detecting genomic islands using bioinformatics approaches. Nat. Rev. Microbiol..

[2] Dobrindt U, Hochhut B, Hentschel U, Hacker J (2004). Genomic islands in pathogenic and environmental microorganisms. Nat. Rev. Microbiol..

[3] Hacker J, Carniel E (2001). Ecological fitness, genomic islands and bacterial pathogenicity. EMBO Rep..

[4] Hacker J, Kaper JB (2000). Pathogenicity islands and the evolution of microbes. Annu. Rev. Microbiol..

[5] Kado CI (2009). Horizontal gene transfer: sustaining pathogenicity and optimizing host–pathogen interactions. Mol. Plant Pathol..

[6] Ho Sui SJ, Fedynak A, Hsiao WW, Langille MG, Brinkman FS (2009). The association of virulence factors with genomic islands. PLoS One.

[7] Juhas M, van der Meer JR, Gaillard M, Harding RM, Hood DW, Crook DW (2009). Genomic islands: tools of bacterial horizontal gene transfer and evolution. FEMS Microbiol. Rev..

[8] Tumapa S, Holden MT, Vesaratchavest M, Wuthiekanun V, Limmathurotsakul D, Chierakul W, Feil EJ, Currie BJ, Day NP, Nierman WC (2008). Burkholderia pseudomallei genome plasticity associated with genomic island variation. BMC Genomics.

[9] Langille MG, Brinkman FS (2009). IslandViewer: an integrated interface for computational identification and visualization of genomic islands. Bioinformatics.

[10] Langille MG, Hsiao WW, Brinkman FS (2008). Evaluation of genomic island predictors using a comparative genomics approach. BMC Bioinformatics.

[11] Waack S, Keller O, Asper R, Brodag T, Damm C, Fricke WF, Surovcik K, Meinicke P, Merkl R (2006). Score-based prediction of genomic islands in prokaryotic genomes using hidden markov models. BMC Bioinformatics.

[12] Hsiao W, Wan I, Jones SJ, Brinkman FS (2003). IslandPath: aiding detection of genomic islands in prokaryotes. Bioinformatics.

[13] Lee CC, Chen YP, Yao TJ, Ma CY, Lo WC, Lyu PC, Tang CY (2013). GI-POP: a combinational annotation and genomic island prediction pipeline for ongoing microbial genome projects. Gene.

[14] Hasan MS, Liu Q, Wang H, Fazekas J, Chen B, Che D (2012). GIST: genomic island suite of tools for predicting genomic islands in genomic sequences. Bioinformation.

[15] Wang H, Fazekas J, Booth M, Liu Q, Che D (2011). An integrative approach for genomic island prediction in prokaryotic genomes. Bioinform. Res. Appl..

[16] Che D, Hasan MS, Wang H, Fazekas J, Huang J, Liu Q (2011). EGID: an ensemble algorithm for improved genomic island detection in genomic sequences. Bioinformation.

[17] Wei W, Guo FB (2011). Prediction of genomic islands in seven human pathogens using the Z-island method. Genet. Mol. Res..

[18] Langille MG, Laird MR, Hsiao WW, Chiu TA, Eisen JA, Brinkman FS (2012). MicrobeDB: a locally maintainable database of microbial genomic sequences. Bioinformatics.

[19] Krzywinski M, Schein J, Birol İ, Connors J, Gascoyne R, Horsman D, Jones SJ, Marra MA (2009). Circos: an information aesthetic for comparative genomics. Genome Res..

[20] Chen L, Xiong Z, Sun L, Yang J, Jin Q (2012). VFDB 2012 update: toward the genetic diversity and molecular evolution of bacterial virulence factors. Nucleic Acids Res..

[21] Liu B, Pop M (2009). ARDB—antibiotic resistance genes database. Nucleic Acids Res..

